# Evaluation of Four Lateral Flow Assays for the Detection of *Legionella* Urinary Antigen

**DOI:** 10.3390/microorganisms9030493

**Published:** 2021-02-26

**Authors:** Alicia Y. W. Wong, Alexander T. A. Johnsson, Aina Iversen, Simon Athlin, Volkan Özenci

**Affiliations:** 1 Division of Clinical Microbiology, Department of Laboratory Medicine, Karolinska Institutet, 141 86 Stockholm, Sweden; aina.iversen@sll.se; 2 Department of Clinical Microbiology, Karolinska University Hospital, Huddinge, 141 86 Stockholm, Sweden; alexander.johnsson@sll.se; 3 Department of Clinical Microbiology, Karolinska University Hospital, Solna, 171 76 Stockholm, Sweden; 4 School of Medical Sciences, Faculty of Medicine and Health, Örebro University, 701 82 Örebro, Sweden; simon.athlin@oru.se

**Keywords:** *Legionella pneumophila*, *Legionella* antigen, pneumonia, urinary antigen test (UAT), BinaxNOW, ImmuView, STANDARD F, Sofia

## Abstract

Urinary antigen tests (UATs) are often used to diagnose Legionnaires’ disease as they are rapid and easy to perform on readily obtainable urine samples without the need for specialized skills compared to conventional methods. Recently developed automated readers for UATs may provide objective results interpretation, especially in cases of weak result bands. Using 53 defined patient urine samples, we evaluated the performance of the BinaxNOW *Legionella* Antigen Card (Abbott), ImmuView *S. pneumoniae* and *Legionella* (SSI Diagnostica), STANDARD F *Legionella* Ag FIA (SD Biosensor), and Sofia *Legionella* FIA (Quidel) simultaneously with their respective automated readers. Automatic and visual interpretation of result bands were also compared for the immunochromatography-based BinaxNOW and ImmuView UATs. Overall sensitivity and specificity of *Legionella* UATs were 53.9–61.5% and 90.0–94.9%, respectively. All four UATs successfully detected all samples from *L. pneumophila* serogroup 1-positive patients, but most failed to detect samples for *Legionella* spp., or other serogroups. Automatic results interpretation of results was found to be mostly concordant with visual results reading. In conclusion, the performance of the four UATs were similar to each other in the detection of *Legionella* urinary antigen with no major difference between automated or visual results reading.

## 1. Introduction

Legionnaires’ disease is an uncommon but severe pneumonia-type illness caused by *Legionella* spp. In 2018, there were almost 10,000 cases of Legionnaire’s disease in the USA [1] and 10,672 confirmed cases in the EU/EEA [2]. It is estimated that Legionnaires’ disease is fatal in around 1 in 10 cases [3,4]. Although culture of clinical lower respiratory tract specimens remains the gold standard method for diagnosis of Legionnaires’ disease, the most commonly utilized diagnostic tool is the use of urinary antigen tests (UATs). Unlike culture-based methods that require 3–5 days and requires training in *Legionella* colony identification, and the use of specific procedures and specialized media for isolating *Legionella* from patient sputum, UATs are easy to perform and results are obtained within 15–20 min, hence speeding up patient diagnosis [5]. It is also common to detect *Legionella* infection by PCR [5]. However, lab staff performing PCR must be specially trained for molecular techniques, and obtaining sputum samples for culture and/or PCR is more challenging compared to the more readily available urine.

There are currently 58 species and more than 70 serogroups known of *Legionella*, of which 30 species have been reported to cause human infection [4]. The vast majority of UATs are focused on detecting *Legionella pneumophila* serogroup 1, which is the causative agent for 50–80% of Legionnaires’ disease cases [5]. There are also a few UATs reported in literature that are not confined to detecting only *L. pneumophila* serogroup 1 [6,7,8,9,10].

Hitherto published studies on rapid *Legionella* UATs for detection of *L. pneumophila* serogroup 1 reported sensitivities and specificities ranging between 55.5–96.0% and 95.6–100%, respectively, using different technologies for antigen detection [8,9,10,11,12,13]. Immunochromatography (ICT)-based UATs produce a visible result band for interpretation, while fluorescent immunoassay (FIA)-based UATs require the use of an automated reader for interpretation. There is a possibility of obtaining visually faint result bands on ICT-based UATs that could be easily missed [7], which could lead to false-negative results.

Automated readers for ICT-based UATs that have been developed recently may provide more objective readings particularly in the case of weak result bands. To our knowledge, the potential benefits of using automated readers for ICT-based UATs has not been yet explored, hence the present study aims to compare the performance of four *Legionella* UATs with automated reading on urine samples from patients with and without PCR-verified *Legionella* infection. Here, we compare two ICT-based UATs, the BinaxNOW *Legionella* Antigen Card (BinaxNOW) (Abbott, Chicago, IL, USA) and ImmuView *S. pneumoniae* and *L. pneumophila* (ImmuView) (SSI Diagnostics, Hillerød, Denmark), and two FIA-based UATs, the STANDARD F *Legionella* FIA (STANDARD F) (SD Biosensor, Gyeonggi, Korea) and Sofia *Legionella* FIA (Sofia) (Quidel Corporation, San Diego, CA, USA) were evaluated simultaneously. In addition, the automatically read results obtained with ICT-based UATs were also compared to visually read results.

## 2. Materials and Methods

### 2.1. Samples

**Figure 1:**
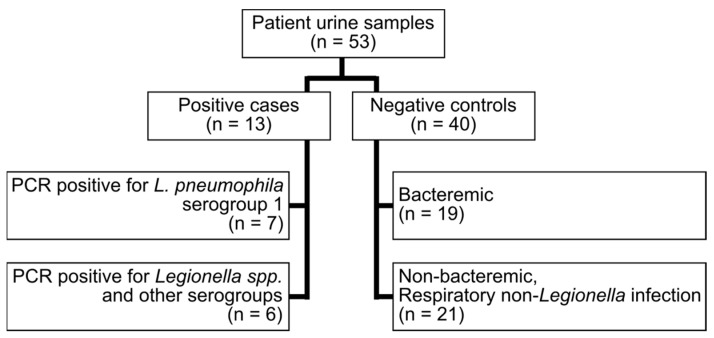
Patient urine samples included in the study.

Urine samples from adult patients (≥18 years old) were collected at the Department of Clinical Microbiology at Karolinska University Hospital, Sweden and the Department of Laboratory Medicine at Örebro University Hospital, Sweden. Urine samples from patients who were positive for *Legionella* by PCR were used as positive cases. Urine samples from patients that had infections of non-*Legionella* etiology were used as negative controls (Figure 1, Tables S1 and S2). All samples were anonymized after culture results had been retrieved. Samples were stored at −20 °C or colder and were thawed in room temperature prior to testing. One sample per patient were used and each sample was tested with all four UATs (BinaxNOW, ImmuView, STANDARD F, and Sofia).

### 2.2. Urinary Antigen Tests

UATs from the four manufacturers were used together with their respective readers. The four UATs included in the present study were the BinaxNOW together with the DIGIVAL instrument, the ImmuView together with the ImmuView Reader, the STANDARD F together with the STANDARD F200 Analyzer, and the Sofia together with the Sofia Fluorescent Immunoassay Analyzer. For the BinaxNOW, STANDARD F, and Sofia, samples were incubated and read by the readers on “Walk Away” mode. UAT setup procedures are briefly described as follows and interpretations were performed according to each manufacturer’s instructions. BinaxNOW setup included dipping the included swab into the sample, inserting it into the sample card, adding two drops of kit-included reagent buffer, and then inserting the sample card into the reader for incubation and interpretation as per manufacturer’s instructions. For ImmuView, three drops of sample were added to the included polypropylene tube followed by addition of two drops of the kit-included reagent buffer, and gently mixed. The UAT strip was then inserted into the tube, and incubation was timed manually before the UAT was inserted into the reader for interpretation. Sample incubation time was 15 min for both the BinaxNOW and ImmuView. Test results for BinaxNOW and ImmuView were also visually interpreted by two independent researchers. The appearance of a correctly colored band that was visible to the naked eye was considered as a positive result. If the visual interpretations of the UAT differed between researchers, the result that was consistent with automated reading was used. For STANDARD F and Sofia, the protocol had no reagent buffer thus only the sample was introduced to the sample well on the cassette using the kit-included fixed volume dropper, and the cassettes were then inserted into their respective reader for incubation and interpretation as per manufacturers’ instructions. Samples were run simultaneously with each of the four UATs, and the results provided by the readers were noted. Sample incubation time was 15 min for STANDARD F, and 10 min for the Sofia. The readers indicated if the sample result was positive or negative for *Legionella* and if the test was valid. If a result obtained for a sample was invalid or the reader displayed an error message, the sample was re-analyzed using a new UAT. The STANDARD F200 Analyzer also indicated the cut-off index (COI) value along with the sample result.

### 2.3. Ethical Permission

The samples that were used in the study were leftover samples that were submitted to the laboratory. The samples were anonymized and information on patient characteristics was not used. Therefore ethical permission was not needed for the study.

### 2.4. Statistical Analysis

Sensitivities and specificities of the UATs were determined, and the confidence intervals (CI) were computed using the Wilson–Brown method. Statistical calculations for the Wilson–Brown method were performed using Prism 8 (GraphPad Software LLC, San Diego, CA, USA). A CI of 95% was used for statistical precision.

## 3. Results

### 3.1. Sample Characteristics

A total of 53 samples were analyzed, 13 were positive cases and 40 negative controls (Figure 1, Tables S1 and S2).

### 3.2. Sensitivity of Legionella UATs

All UATs successfully detected *Legionella* antigen in samples from four patients with confirmed *L. pneumophila* serogroup 1 infection, and three patients with non-serogrouped *L. pneumophila* (Table 1). The UATs did not detect *Legionella* antigen in samples from patients infected with other *Legionella* spp. (Table 2, Table S1). One sample from a patient with PCR-confirmed *L. pneumophila* serogroup 6 was detected by ImmuView only and yielded an invalid result with Sofia.

### 3.3. Specificity of Legionella UATs

The specificities of the four *Legionella* UATs ranged from 90.0–94.9% (Table 3). A total of six negative controls yielded false positive results in one or more UATs (Table 4). For one sample (N15, Table 4, Table S2), the reader indicated insufficient sample volume error in all four attempts with the STANDARD F UAT. These cassettes from four failed attempts were inspected, and it was observed that the sample failed to reach the end of the test strip in the cassettes.

### 3.4. Agreement between Visual and Automatic Reading of UATs

**Figure 2:**
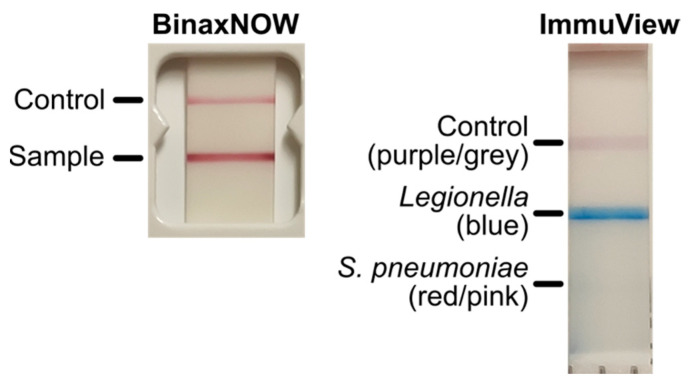
BinaxNOW and ImmuView test results for positive case P4.

The visual results interpretations of the BinaxNOW and ImmuView UATs by the two researchers were 100% in agreement with each other. However, there were some inconsistencies observed between the visual and automatic reading results in two of the samples with the BinaxNOW UAT and with the ImmuView UAT. For the BinaxNOW UAT, two samples (N8 and N15, Table 4, Table S2) yielding a positive result by automatic reading were interpreted as negative visually by the two researchers. For the ImmuView UAT, one *Legionella* positive sample (P4, Table S1) also yielded a positive result for *S. pneumoniae* on the same test strip by automatic reading, but was negative by visual. As this urine sample was tested, both BinaxNOW and ImmuView yielded very strong test results for *Legionella* antigen, which may have interfered with the automated interpretation of pneumococcal antigen detection by the ImmuView reader (Figure 2). Another sample, used as a negative control (N14, Table 4, Table S2), yielded an invalid visual test result with the ImmuView as both the test line for *Legionella* and the control line turned purple, while the automatic reading yielded a positive result. When the sample was re-analyzed, the visual test result was negative while the automatic result was positive.

## 4. Discussion

Using a rapid UAT for diagnosing Legionnaires’ disease is recommended by pneumonia management guidelines due to the simplicity to perform the test on urine samples. The assay turn-around time is within minutes, with high accuracy in detecting *L. pneumophila* serogroup 1 antigen in previous studies [5]. Therefore, it is utterly important to compare the available commercial UAT methods for detection of *Legionella* spp. in urine. The present study compares for the first time four UATs using clinical samples with their respective automated interpretation, showed that the UATs performed similarly to each other. All UATs examined detected *Legionella* urinary antigen in samples from patients with *L. pneumophila* serogroup 1 infection. The specificity of the UATs were 90–95%. In addition, results were found to be mostly concordant between automatic and visual interpretation of the BinaxNOW and ImmuView UATs.

All four UATs yielded negative results on samples that were non-*L. pneumophila* serogroup 1 *Legionella* spp. positive, which is in line with the test validation by the manufacturers, and has been demonstrated in previous studies [7,8]. As for the detection of other *L. pneumophila* serogroups, a sample from a patient infected with *L. pneumophila* serogroup 6 tested positive in the ImmuView despite the detection of other serogroups was not explicitly stated in the ImmuView product information. The BinaxNOW UAT product information has stated that the UAT is unable to detect urinary antigens of other *L. pneumophila* serogroups or *Legionella* spp., although past studies have shown that it is capable of detecting urinary antigens of other *L. pneumophila* serogroups [7,8]. In contrast, the product information for the STANDARD F and Sofia UATs mentioned the potential for both to detect the antigens of other *L. pneumophila* serogroups (serogroups 3, 5, 6, and 8 for the STANDARD F; serogroups 3, 4, and 6 for the Sofia). However, the *L. pneumophila* serogroup 6 sample tested negative on both UATs in the present study. Nevertheless, the detection non-*L. pneumophila* serogroup 1 infection is unreliable and underscores the importance of not relying solely on UATs for diagnosis of Legionnaires’ disease as they would miss 20–50% of Legionnaires’ disease cases that are caused by other *Legionella* species and serogroups [5].

The specificities of the UATs were found to be slightly lower compared to what was reported in other studies, perhaps due to the small sample size. Interestingly, we observed that 5/6 of the samples that tested false positive in one or more of the UATs were from patients with Gram-negative bacteremia. To our knowledge, false positivity of UATs in cases of non-*Legionella* bacteremia has not been previously reported. The underlying reason for this finding is not known. The last false positive result was a sample from a patient without bacteremia but was respiratory culture positive for *C. albicans* tested positive in all four UATs. This patient’s bronchoalveolar lavage sample was PCR-negative for *Legionella*. As false positivity may occur if the patient had a past *Legionella* infection since *Legionella* antigens can be excreted in urine for as long as a year [14], it is not possible to exclude the possibility of resolved any past *Legionella* infection that could give rise to antigen positivity in the urine. False positivity with *Legionella* UATs have also been previously demonstrated in patients with serum sickness [15,16], and for samples of this nature, boiling is a possible way to eliminate false positives due to the heat sensitive nature of rheumatoid-like factors compared to the heat resistance of bacterial antigens [16]. Contradictory evidence for boiling of samples exist in the literature. For instance, it was shown that boiling samples increased the specificity of the Sofia UAT by 2.3% (compared to results obtained by BinaxNOW) [17]; however, another study showed that while boiling did not affect the sensitivity of the ImmuView UAT, the sensitivity of the BinaxNOW was worse in boiled samples compared to unboiled samples tested for *Legionella* antigen [7]. Sample boiling to confirm positive results is recommended by some manufacturers of UATs, but was not recommended by the BinaxNOW and STANDARD F UATs. In the present study, all samples were tested by all four tests in similar conditions, therefore, the urine samples were not boiled.

The interpretation of colorimetric bands on ICT-based assays could be subjective compared to FIA-based assays that are read automatically [17]. In the present study, 3 out of 106 total readings of ICT-based UAT results obtained on automated readers were in line with visually read results. In addition, one of the negative control samples (N6, Table 4, Table S2) that tested positive for *Legionella* with the ImmuView UAT by both automatic reading and visually was noted to have a visually faint *Legionella* band by both researchers. While this was not judged as an inconsistency of results between automated and visual readings, it indicated that automated readers could be more sensitive in detecting faint result bands that otherwise might be missed if only a visual reading was done.

The STANDARD F *Legionella* UAT was unique in this study as the only UAT that presents a COI value as a way to quantify the concentration of *Legionella* antigen in the sample in addition to the dichotomous test results (positive or negative). As none of the other readers provided quantifiable results, we were unable to compare signal intensity across the four UATs. The importance of the COI value in clinical routine has not been studied, and there are no previous studies using the STANDARD F for *Legionella* antigen detection.

Other aspects that were observed in the present study include the occurrence of invalid test results or problems with running the UAT that could not be resolved on two of the samples with FIA-based UATs. The sample that received an insufficient volume error with the STANDARD F UAT received the same error despite testing on new UAT cassettes as recommended by the manufacturer instructions. The manufacturer guidelines were given for errors in general, and did not have any further instructions on what could be done for this particular type of error. The sample did not appear to be viscous which would have affected the sample diffusion on the UAT. Additionally, the sample could be tested on the other UATs, and no issues were encountered when the same sample was tested separately on the STANDARD F *S. pneumoniae* UAT; hence, the reason for this occurrence is unknown. Additionally, this is the first time where all UATs were evaluated with their respective readers, and we observed that automated and visual interpretation of ICT-based UATs were mostly consistent with each other. For the ImmuView, we also noted that the samples with the strong positive *Legionella* signal interfered with the automated interpretation of pneumococcal antigen detection, resulting in a false positive result for pneumococcal antigen. This may be something that needs to be solved to avoid false positive results.

Information on how various UATs are utilized under clinical testing conditions are an important aspect to enable clinics to decide which UAT is most appropriate to implement in their diagnostic workflow with consideration of their specific needs and available resources. However, this is often not reported in UAT comparison studies. In the present study, comparing four UATs simultaneously, we had the unique opportunity to compare their user friendliness, and in this respect we observed that the FIA-based UATs have fewer steps required to setup samples, and hence less hands-on time. Despite requiring more sample setup procedures, the ICT-based UATs do have the flexibility to not require a reader for interpreting results. For the BinaxNOW, STANDARD F, and Sofia UATs, the availability of barcode scanners, connection to the laboratory information system (LIS), as well as the flexibility to choose between automated incubation followed by automated reading of samples, or reading of multiple samples in quick succession after a manually timed incubation also provides increased convenience for staff.

A limitation to our study is that we used frozen urine samples for evaluating UAT performance, and that sample numbers were small. We experienced difficulty obtaining more positive samples due to the current SARS-COV-2 pandemic. Another limitation is that the information we were able to retrieve from the LIS was limited to recent blood and respiratory culture results in relation to the urine sample, hence making it difficult to interpret the false positive results obtained.

In conclusion, we compared four *Legionella* UATs with automatic interpretation of test results using defined clinical samples and found that they performed similarly in detection of *Legionella* urinary antigen. Results between automated and visual interpretation of ICT-based UATs were mostly consistent with each other. The use of an automated reader also brings increased flexibility and convenience in performing urinary antigen assays in the clinical routine.

## Figures and Tables

**Table 1:** Sensitivity of four *Legionella* urine antigen assays on samples *.

Assay	Positive Result	Negative Result	Sensitivity (95% CI)
**BinaxNOW**	7	0	100.0 (64.6–100.0)
**ImmuView**	7	0	100.0 (64.6–100.0)
**STANDARD F**	7	0	100.0 (64.6–100.0)
**Sofia**	7	0	100.0 (64.6–100.0)

* Urine samples from patients with PCR-confirmed *L. pneumophila* infection.

**Table 2:** Positivity of four *Legionella* urine antigen assays on samples from patients with PCR-confirmed *Legionella* infection by species other than *Legionella pneumophila* serogroup 1 *.

Assay	Positive Result	Negative Result	Invalid/Error	Positivity Rate (95% CI)
**BinaxNOW**	0	6	0	0.0 (0.0–39.0)
**ImmuView**	1	5	0	16.7 (0.9–56.4)
**STANDARD F**	0	6	0	0.0 (0.0–39.0)
**Sofia**	0	5	1	0.0 (0.0–39.0)

* L. longbeachae (n = 2), L. bozemanii (n = 2), L. pneumophila serogroup 6 (n = 1), Legionella spp. (n = 1).

**Table 3:** Specificities of four *Legionella* urine antigen assays.

Assay	Positive Result	Negative Result	Invalid/Error	Specificity (95% CI)
**BinaxNOW**	3	37	0	92.5 (80.1–97.4)
**ImmuView**	4	36	0	90.0 (77.0–96.0)
**STANDARD F**	2	37	1	94.9 (83.1–99.1)
**Sofia**	3	37	0	92.5 (80.1–97.4)

**Table 4:** False positive and discordant test results in six patients without *Legionella* infection.

Sample	Blood Culture Result	Respiratory Culture Result	BinaxNOW	ImmuView	STANDARD F	Sofia
N6	*B. fragilis*	Negative (normal flora)	Negative	Positive	Negative	Negative
N7	*E. coli*	Negative (normal flora)	Negative	Positive	Positive	Positive
N8	*E. coli*	Negative (normal flora)	Positive	Negative	Negative	Negative
N14	*K. pneumoniae*	Negative (normal flora)	Negative	Positive	Negative	Positive
N15	*P. mirabilis*	Negative (normal flora)	Positive	Negative	Insufficient volume error	Negative
N20	Negative	*C. albicans*	Positive	Positive	Positive	Positive

## Data Availability

The data presented in this study are openly available upon request.

## Supplementary Materials

The following are available online at https://www.mdpi.com/2076-2607/9/3/493/s1, Table S1: *Legionella* UAT results for *Legionella* positive cases as interpreted by automatic readers, Table S2: *Legionella* UAT results for negative controls as interpreted by automatic readers.
