# Supplementary material for: Evaluation of Four Lateral Flow Assays for the Detection of Legionella Urinary Antigen
Source: Microorganisms. 2021 Feb 26;9(3):493. doi: 10.3390/microorganisms9030493 (PMC7996842; doi:10.3390/microorganisms9030493)
Supplement: Supplementary file 1 [file microorganisms-09-00493-s001.pdf]

microorganisms

MDPI

Supplementary Data

Evaluation of four lateral flow assays for the detection of Legionella urinary antigen

Alicia Y. W. Wong, Alexander T. A. Johnsson, Aina Iversen, Simon Athlin, and Volkan Özenci

Table S1. *Legionella* UAT results for *Legionella* positive cases as interpreted by automatic readers

Sample	Clinical result	BinaxNOW	ImmuView	STANDARD F	Sofia
P1	*L. pneumophila*	Positive	Positive	Positive	Positive
P2	*L. pneumophila*	Positive	Positive	Positive	Positive
P3	*L. pneumophila* (serogroup 1)	Positive	Positive	Positive	Positive
P4	*L. pneumophila* (serogroup 1)	Positive	Positive	Positive	Positive
P5	*L. pneumophila* (serogroup 1)	Positive	Positive	Positive	Positive
P6	*L. pneumophila* (serogroup 1)	Positive	Positive	Positive	Positive
P7	*L. pneumophila* (serogroup 1)	Positive	Positive	Positive	Positive
P8	*L. pneumophila* (serogroup 6)	Negative	Positive	Negative	Invalid
P9	*Legionella spp.*	Negative	Negative	Negative	Negative
P10	*Legionella spp.* (*L. bozemanii*)	Negative	Negative	Negative	Negative
P11	*Legionella spp.* (*L. bozemanii*)	Negative	Negative	Negative	Negative
P12	*Legionella spp.* (*L. longbeachae*)	Negative	Negative	Negative	Negative
P13	*Legionella spp.* (*L. longbeachae*)	Negative	Negative	Negative	Negative

Table S2. *Legionella* UAT results for negative controls as interpreted by automatic readers

Sample	Blood culture result	Respiratory culture result	BinaxNOW	ImmuView	STANDARD F	Sofia
N1	*S. pneumoniae*	*Proteus spp.*	Negative	Negative	Negative	Negative
N2	*S. aureus*	*S. aureus*	Negative	Negative	Negative	Negative
N3	*S. aureus*	*S. aureus*	Negative	Negative	Negative	Negative
N4	Group B *Streptococcus*	*S. aureus* and *M. catarrhalis*	Negative	Negative	Negative	Negative
N5	*S. pneumoniae*	*S. pneumoniae*	Negative	Negative	Negative	Negative
N6	*B. fragilis*	Negative (normal flora)	Negative	Positive	Negative	Negative
N7	*E. coli*	Negative (normal flora)	Negative	Positive	Positive	Positive
N8	*E. coli*	Negative (normal flora)	Positive	Negative	Negative	Negative
N9	*E. coli*	Negative (normal flora)	Negative	Negative	Negative	Negative
N10	*E. coli*	Negative (normal flora)	Negative	Negative	Negative	Negative
N11	*E. coli*	Negative (normal flora)	Negative	Negative	Negative	Negative
N12	*E. faecalis*	Negative (normal flora)	Negative	Negative	Negative	Negative
N13	*E. faecalis*	Negative (normal flora)	Negative	Negative	Negative	Negative
N14	*K. pneumoniae*	Negative (normal flora)	Negative	Positive	Negative	Positive
N15	*P. mirabilis*	Negative (normal flora)	Positive	Negative	Insufficient volume error	Negative
N16	*P. mirabilis*	Negative (normal flora)	Negative	Negative	Negative	Negative
N17	*S. aureus*	Negative (normal flora)	Negative	Negative	Negative	Negative
N18	*S. pneumoniae*	Negative (normal flora)	Negative	Negative	Negative	Negative
N19	*E. coli*	No result	Negative	Negative	Negative	Negative
N20	Negative	*C. albicans*	Positive	Positive	Positive	Positive
N21	Negative	*H. influenzae*	Negative	Negative	Negative	Negative
N22	Negative	*H. influenzae*	Negative	Negative	Negative	Negative
N23	Negative	*H. influenzae*	Negative	Negative	Negative	Negative
N24	Negative	*H. influenzae*	Negative	Negative	Negative	Negative
N25	Negative	*K. pneumoniae*	Negative	Negative	Negative	Negative
N26	Negative	*M. catarrhalis*	Negative	Negative	Negative	Negative
N27	Negative	*M. catarrhalis*	Negative	Negative	Negative	Negative
N28	Negative	*M. catarrhalis*	Negative	Negative	Negative	Negative
N29	Negative	*M. catarrhalis*	Negative	Negative	Negative	Negative
N30	Negative	*M. catarrhalis*	Negative	Negative	Negative	Negative
N31	Negative	*M. catarrhalis*	Negative	Negative	Negative	Negative
N32	Negative	*M. catarrhalis*	Negative	Negative	Negative	Negative
N33	Negative	*M. catarrhalis*	Negative	Negative	Negative	Negative

N34	No result	*M. tuberculosis*	Negative	Negative	Negative	Negative
N35	Negative	Negative (PCR: SARS-COV-2)	Negative	Negative	Negative	Negative
N36	Negative	*S. aureus*	Negative	Negative	Negative	Negative
N37	Negative	*S. pneumoniae*	Negative	Negative	Negative	Negative
N38	Negative	*S. pneumoniae*	Negative	Negative	Negative	Negative
N39	Negative	*S. pneumoniae*	Negative	Negative	Negative	Negative
N40	Negative	Unspecified yeast	Negative	Negative	Negative	Negative
